# FISH-based mitotic and meiotic diakinesis karyotypes of *Morus notabilis* reveal a chromosomal fusion-fission cycle between mitotic and meiotic phases

**DOI:** 10.1038/s41598-017-10079-6

**Published:** 2017-08-29

**Authors:** Yahui Xuan, Chaoshuo Li, Yue Wu, Bi Ma, Ruiling Liu, Zhonghuai Xiang, Ningjia He

**Affiliations:** State Key Laboratory of Silkworm Genome Biology, Southwest University, Chongqing, 400715 China

## Abstract

Mulberry (*Morus spp*.), in family Moraceae, is a plant with important economic value. Many polyploid levels of mulberry have been determined. In the present study, the fluorescence *in situ* hybridization (FISH) technique was applied in *Morus notabilis*, using four single-copy sequences, telomere repeats, and 5S and 25S rDNAs as probes. All the mitotic chromosomes were clearly identified and grouped into seven pairs of homologous chromosomes. Three dot chromosome pairs were distinguished by the FISH patterns of the 25S rDNA probe and a simple sequence repeat (SSR2524). According to the FISH signals, chromosome length and morphology, detailed meiotic diakinesis karyotype was constructed. Interestingly, only six bivalent chromosomes were observed in diakinesis cells. The 25S rDNA probe was used to illustrate chromosome alterations. The results indicated that mitotic chromosomes 5 and 7 fused into diakinesis chromosome 5 during the meiotic phase. In mitotic cells, the fused chromosome 5 broke into chromosomes 5 and 7. A chromosomal fusion-fission cycle between the meiotic and mitotic phases in the same individual is reported here for the first time. This finding will contribute to the understanding of karyotype evolution in plants.

## Introduction

Mulberry (*Morus spp*.), in family Moraceae, is a deciduous woody plant of great economic importance that is distributed worldwide. Leaves of mulberry are the main food for silkworms^1^, and the delicious and nutritious mulberry fruit is a functional food^2^. Mulberry also has great medical value owing to the presence of many bioactive components^3^. Mulberry has the abundant resources of ploidy with a series of chromosome numbers, e.g. 14, 28, 35, 42, 56, 84, 112, and 308^4, 5^. Among the resources with known chromosome numbers, mulberry with 2n = 28 is the most commonly found plant. The basic chromosome number (x = 14) of mulberry was proposed a long time ago^6, 7^. This idea has been widely cited in later studies^8–10^. Cytogenetic researchers, Das^11^ and Datta^12^, doubted this theory based on their observations of secondary associations in a few varieties of *Morus indica*. However, their work was not supported until the karyotype of *M. notabilis* was reported^13^. Based on the differences in chromosome morphology and length, the 14 chromosomes of *M. notabilis* were grouped into seven pairs comprising one pair of long chromosomes, two pairs of middle length chromosomes, one pair of short chromosomes, and three pairs of dot chromosomes. As a result, a basic chromosome number of seven was confirmed but not fully accepted. In mulberries with higher ploidy levels (2n ≥ 28), the chromosomes were found to be small and similar in morphology^8^, which made it impossible to identify all the chromosomes using classical methods. Therefore, new techniques are needed for the further study of mulberry chromosomes.

Fluorescence *in situ* hybridization (FISH) is more powerful than the classical methods and has been used widely in cytogenetic studies of plants, especially in species with small and similar chromosomes^14^. The FISH technique allows chromosomes to be accurately identified by specific DNA sequences based on signal patterns. Many probes have been developed for FISH. The single-copy sequence probes can accurately identify chromosomes^15, 16^. The rDNA probes are the most utilized probes in cytogenetic studies^17–19^. Repeat sequences can provide more information than the other probes, and all the chromosomes have been identified using one repeat sequence probe^20^. Mitotic metaphase chromosomes and pachytene chromosomes have been used widely for chromosome identification, physical mapping, and transgene detection^21, 22^. Meiotic diakinesis chromosomes have been applied in the studies of polyploidy and particular aspects of chromosomes. However, diakinesis chromosomes have not been systemically applied in FISH-based karyotyping^23^.

In this study, FISH-based karyotypes on mitotic and meiotic diakinesis chromosomes of *M. notabilis* were determined. All the individual chromosomes were identified by four single-copy sequence probes, and 5S and 25S rDNA probes. The 25S rDNA probe was also used to trace the dynamics of mitotic chromosomes and the different stages of meiotic chromosomes. Here, we describe a chromosomal fusion–fission cycle between the mitotic and meiotic phases in the same individual *M. notabilis* for the first time.

## Results

### FISH-based mitotic karyotype of *M. notabilis*

**Figure 1:**
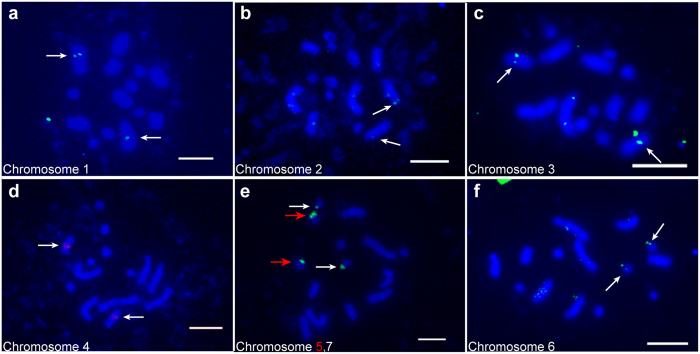
FISH mapping of single-copy sequence probes, and 25S and 5S rDNA probes on mitotic metaphase chromosomes of *M. notabilis* with DAPI counterstaining (**a**) Chromosome 1 located by probe morus027496 (green). (**b**) Chromosome 2 located by probe morus027717 (green). (**c**) Chromosome 3 located by probe morus026579 (green). (**d**) Chromosome 4 located by probe 5S rDNA (red). (**e**) Chromosome 5 located by probe 25S rDNA (green) with a pair of bright signals (red arrow) and chromosome 7 with a pair of minor signals (white arrow). (**f**) Chromosome 6 located by probe SSR2524 (green). Arrows indicated the FISH signals of the probes. Scale bars represent 5 μm.

The mitotic cells of *M. notabilis* contain 14 chromosomes grouped into seven pairs^13^. We used the FISH technique to further examine the karyotype of *M. notabilis*, especially to identify the two pairs of middle length chromosomes and three pairs of dot chromosomes. All the chromosomes were located by at least one of the probes. Table 1 summarizes the information for four out of the six single-copy sequence probes, and the 5S and 25S rDNA probes used in this study. The longest chromosome, chromosome 1, was located by probe morus027496 (Fig. 1a). The two middle length chromosomes, chromosomes 2 and 3, were located by probes morus027717 and morus026579, respectively. The morus027717 mapped onto the distal part of chromosomes 2 (Fig. 1b,c). Chromosome 4 was recognized by the 5S rDNA probe (Fig. 1d). The 25S rDNA probe mapped onto two of the three dot chromosomes. Based on minor morphology differences and signal intensities, we named these two dot chromosomes as chromosomes 5 and 7 since chromosome 5 was longer and had a larger signal than chromosome 7, the smallest chromosome (Fig. 1e). The remaining dot chromosome, chromosome 6, was distinguished by the location of probe SSR2524, which was named after the simple sequence repeat (SSR) 2524 locus (Fig. 1f).

**Table 1:** Probes used for chromosome identification in *M. notabilis*.

Mitotic chromosome	Probe	Length of probe (bp)	Length of subclones (bp)
1	morus027496	11,510	2,792; 3,043; 3,132; 2,543
2	morus027717	12,897	2,796; 2,872; 1,722; 1,740
3	morus026579	10,567	1,279; 1,783; 1,787; 1,988; 1,957; 1,773
4	5S rDNA	673	/
5	25S rDNA	772	/
6	SSR2524	10,285	3,100; 1,154; 1,539; 1,553; 1,446; 1,493
7	25S rDNA	772	/

### FISH-based diakinesis karyotype of *M. notabilis*

**Figure 2:**
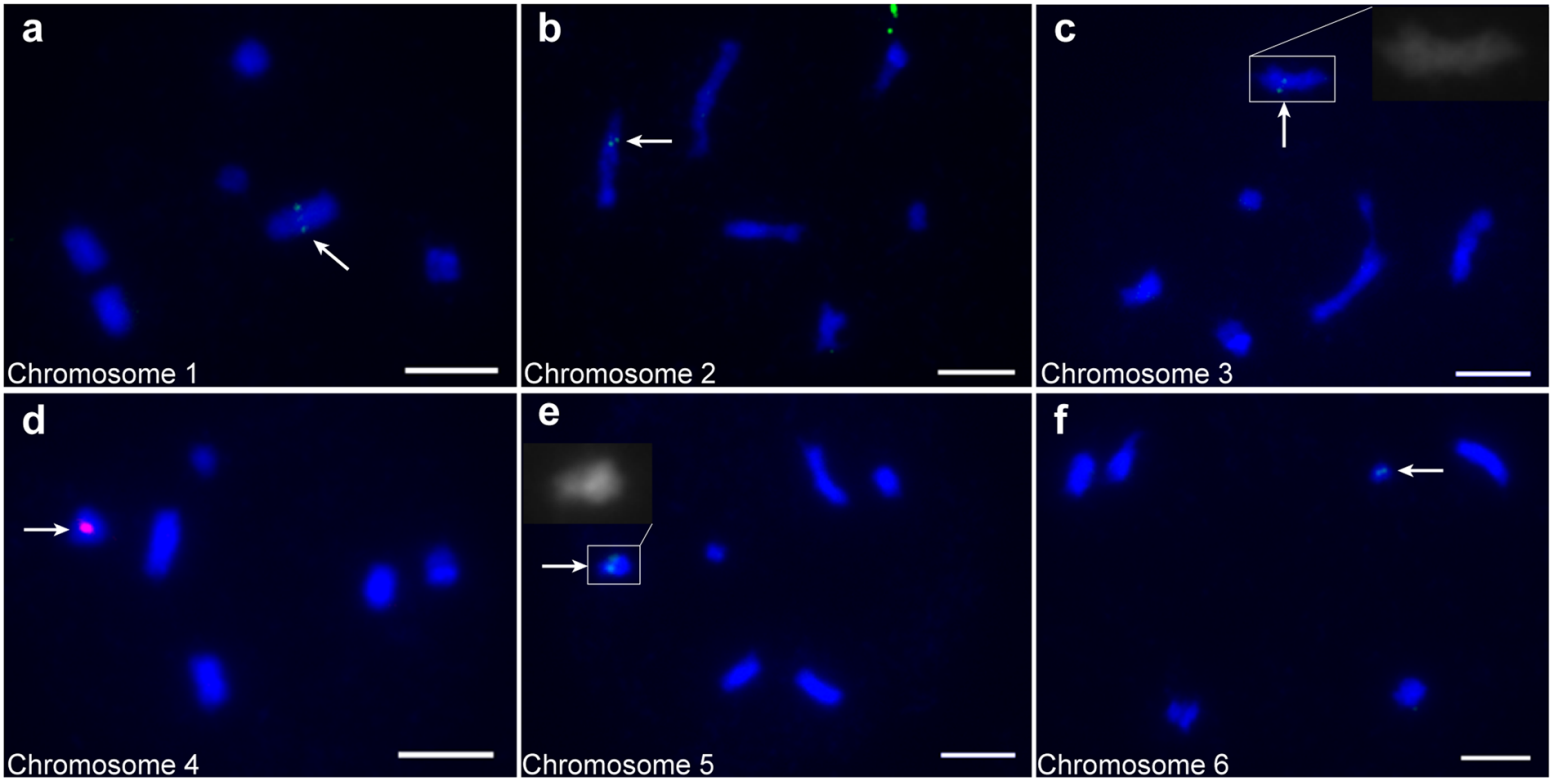
FISH mapping of single-copy sequence probes, and 25S and 5S rDNA probes on diakinesis chromosomes of *M. notabilis* with DAPI counterstaining (**a**) Chromosome 1 located by probe morus027496 (green). (**b**) Chromosome 2 located by probe morus027717 (green). (**c**) Chromosome 3 located by probe morus026579 contains a distinct primary constriction. The inset showed a two times larger magnification of the chromosome. (**d**) Chromosome 4 located by probe 5S rDNA (red). (**e**) Chromosome 5 located by probe 25S rDNA (green) had a special morphology, which suggested the short arm may be embedded in the long arm. The inset showed a two-times-larger magnification of the chromosome (**f**) Chromosome 6 located by probe SSR2524 (green). Arrows indicated the FISH signals of the probes. Scale bars represent 5 μm.

Surprisingly, only six fully paired bivalents in the diakinesis phase were found when we observed the meiotic chromosomes of *M. notabilis* (Fig. 2). To get details of the organization of the diakinesis chromosomes, we used all the probes applied above to identify the chromosomes by FISH. In contrast to the results for the mitotic chromosomes, probes morus027496 and 5S rDNA were located on chromosomes 1 and 4, respectively (Fig. 2a,d). Chromosomes 2 and 3 showed distinguishable differences in morphology and length (Table 2). We named the longer chromosome containing the morus027717 locus as chromosome 2 (Fig. 2b), and the other shorter chromosome containing the morus026579 locus and a distinct primary constriction as chromosome 3 (Fig. 2c). The 25S rDNA probe mapped to only one chromosome, which was similar in length to chromosome 4 and contained a distinct morphology, as shown in the inset in Fig. 2e. We named this chromosome containing the 25S rDNA locus as chromosome 5 because it was similar to the largest dot mitotic chromosome in the signal pattern. The SSR probe SSR2524 mapped onto the smallest chromosome, which we named chromosome 6 because of its similarity to mitotic chromosome 6 (Fig. 2f).

**Table 2:** Relative lengths of the diakinesis chromosomes of *M. notabilis*.

Diakinesis chromosome	Chromosome1	Chromosome2	Chromosome3	Chromosome4	Chromosome5	Chromosome6
Relative length (%)	31.85 ± 4.26	20.11 ± 2.04	18.60 ± 1.83	12.25 ± 1.56	11.05 ± 1.59	6.14 ± 1.73

**Figure 3:**
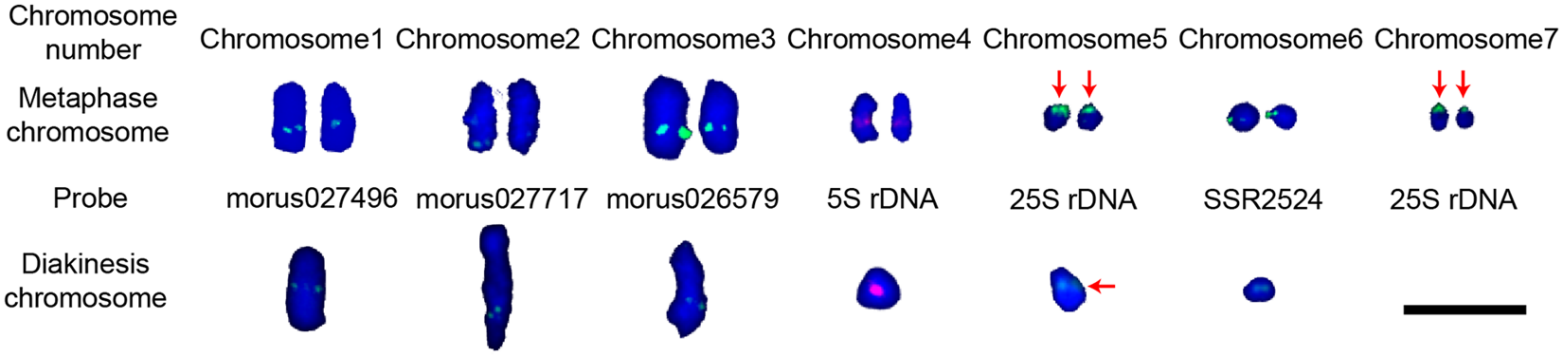
Karyotypes of *M. notabilis* showing seven pairs of mitotic chromosomes and six meiotic chromosomes. The images of the mitotic chromosomes were separated out from the Fig. 1 using Adobe Photoshop CS6. The images of the diakinesis karyotype showing six bivalent chromosomes were separated out from Fig. 2. Chromosomes were ordered based on a combination of chromosome length, morphology, and FISH signal pattern. The red arrows pointed at the fused mitotic chromosome 5 and 7, and the diakinesis chromosome 5. Scale bar represents 5 μm.

To further analyze the diakinesis karyotype, the relative lengths of each chromosome were measured in 30 diakinesis cells in which the chromosomes had been identified (Table 2). Chromosome 1 made up 31.85 ± 4.26% of the total length of all the chromosomes. Chromosomes 2 and 3 had distinguishably different lengths of 20.11 ± 2.04% and 18.60 ± 1.83%, respectively. Chromosomes 4 and 5 had similar lengths (12.25 ± 1.56% and 11.05 ± 1.59%, respectively). Chromosome 6 was the shortest, making up only 6.14 ± 1.73% of the total length. We constructed the final karyotype of *M. notabilis* by combining the morphology and length of diakinesis chromosome and the FISH signal patterns (Fig. 3). The numbering of the chromosomes conformed to the descending order of chromosome length.

### Location patterns of 25S rDNA on meiotic and mitotic chromosomes

**Figure 4:**
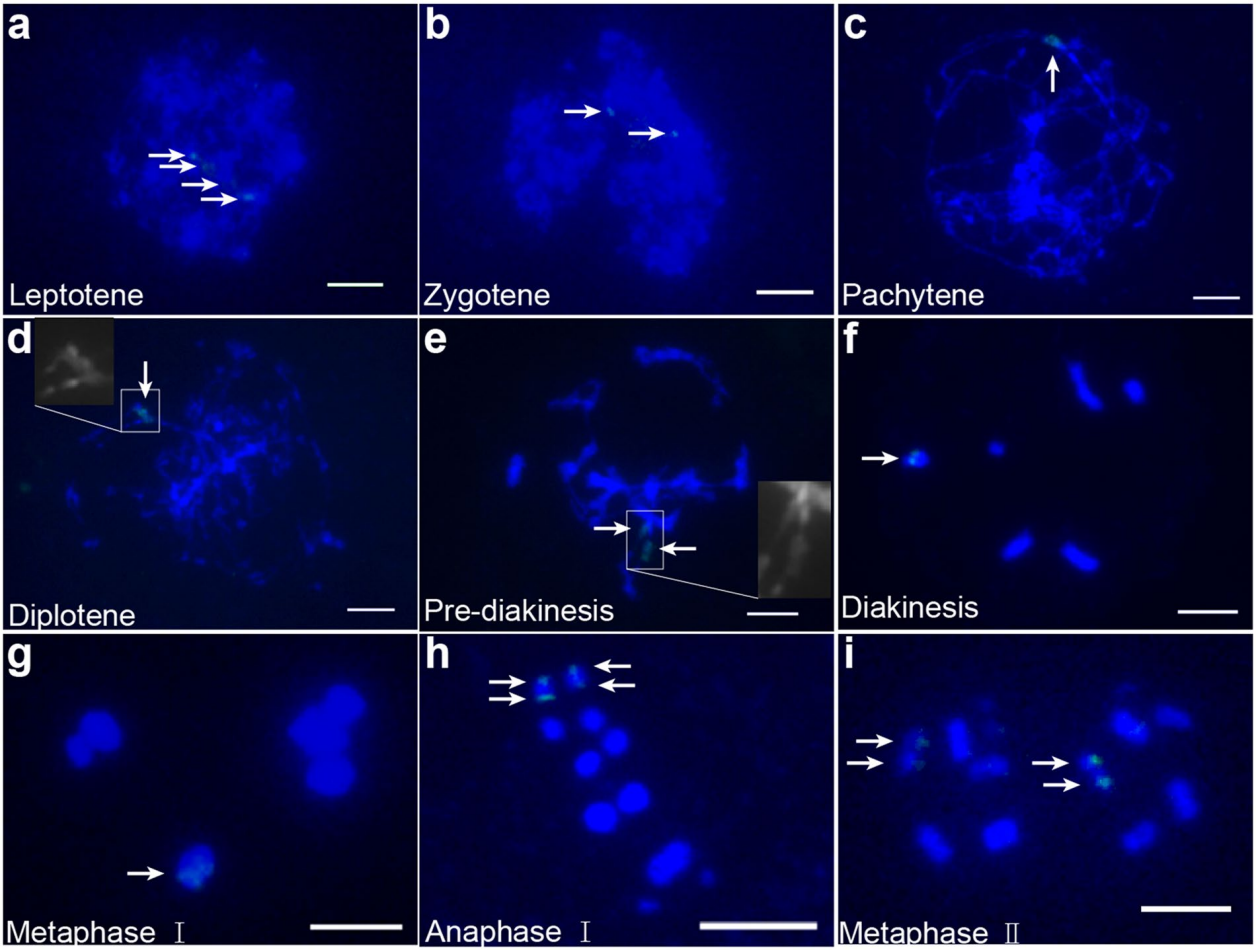
FISH mapping of the 25S rDNA probe location patterns on meiotic chromosomes of *M. notabilis* at different stages with DAPI counterstaining (**a**) Four loci were resolved on the leptotene chromosome. (**b**) Two loci were resolved on the zygotene chromosome. (**c**) One bright locus was resolved on the pachytene chromosome. (**d**) One bright locus was resolved on the diplotene chromosome. The inset showed a two times larger magnification of the region indicated. (**e**) Two loci were resolved on the pre-diakinesis chromosome. The inset showed a two times larger magnification of the region indicated. (**f**) One locus was resolved on the diakinesis chromosome. (**g**) One locus was resolved on the meiotic metaphase I chromosome. (**h**) Two loci were resolved for every 12 chromosomes in meiotic metaphase II. Arrows indicated the FISH signals of the 25S rDNA probe. Scale bars represent 5 μm.

To reveal the mechanism associated with the reduction of the mitotic chromosome complement of seven chromosomes to the complement of six meiotic chromosomes, we traced the pattern of the 25S rDNA probe signals on the meiotic chromosomes. Interestingly, we detected four loci on the leptotene chromosome (Fig. 4a). Because homologous chromosomes were paired, we observed two loci on the zygotene chromosome (Fig. 4b) and, strangely, one large locus on the pachytene chromosome (Fig. 4c). One bright locus was resolved on the diplotene chromosome when the homologous chromosome arms were separated, as shown in the inset. In the pre-diakinesis stage, most chromosomes showed one signal, but some had two clearly separated signals (Fig. 4e). The diakinesis chromosome showed only one locus (Fig. 4f). Finally, one locus was observed on the metaphase I chromosome (Fig. 4g), and two loci were observed on the anaphase I homologous chromosomes and metaphase II sister chromosomes separated from each other (Fig. 4h,i).

**Figure 5:**
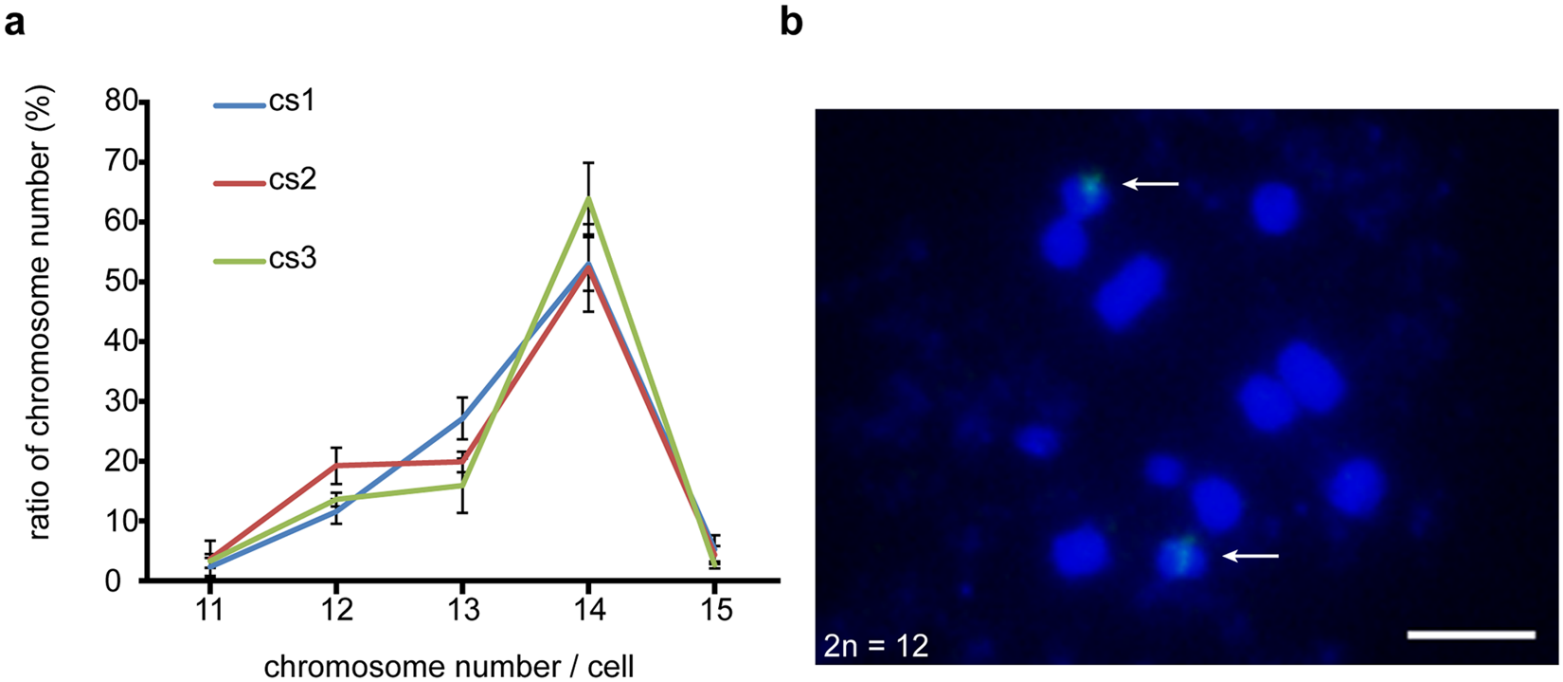
Chromosome numbers and FISH on metaphase chromosomes (2n = 12) using 25S rDNA as probe in wild adult *M. notabilis* trees (**a**) Metaphase chromosome numbers from three wild adult trees were counted in 100 metaphase cells for three replicates. Standard deviations were calculated. The chromosome number of 14 was the most common in all three trees. Only 11–20% of the cells had 2n = 12 metaphase chromosomes. (**b**) A pair of FISH signals from the 25S rDNA probe were located on the 2n = 12 metaphase chromosomes of *M. notabilis*. Arrows indicated the FISH signals of the 25S rDNA probe. Scale bar represents 5 μm.

To determine whether the mitotic chromosome number of 2n = 12 existed, for which the meiotic chromosome number would be doubled, we counted chromosome numbers in 100 metaphase cells of one female and two male wild adult *M. notabilis* trees for three replicates, and took into account only chromosome numbers between 11 and 15. The results are shown in Fig. 5a. We found that the highest chromosome number was 14 and 11–20% of the cells had 2n = 12 metaphase chromosomes. The cells that had 2n = 12 chromosomes were mapped by FISH using the 25S rDNA probe, and two signal loci were found on a pair of chromosomes (Fig. 5b).

### Tel-FISH of *M. notabilis*

**Figure 6:**
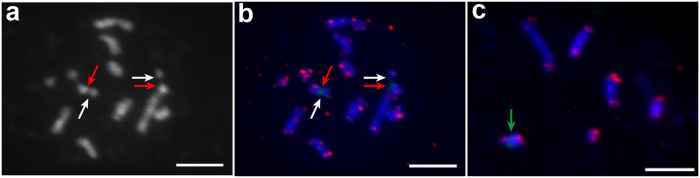
FISH mapping of 25S rDNA (green) and telomere repeats (red) on metaphase chromosomes and diakinesis chromosomes of *M. notabilis* with DAPI counterstaining. (**a**) showed chromosomes in (**b**) with DAPI counterstaining in gray color, the metaphase chromosomes 5 and 7 were indicated by red and white arrows, respectively. (**b**) Red and white arrows indicated the FISH signals of the 25S rDNA probe on metaphase chromosomes 5 and 7, respectively, and (**c**) green arrow indicated the FISH signal on the diakinesis chromosome 5. Scale bars represent 5 μm.

Tel-FISH was performed on mitotic and meiosis chromosomes to track the dynamics of chromosomes. An Arabidopsis-like telomere sequence with (TTTAGGG)_42_ was cloned using the genomic DNA of *M. notabilis* as template. 25S rDNA probe labelled with digoxigenin-11-dUTP was used as landmark of chromosomes 5 and 7 (Fig. 6a,b. red arrows for chromosome 5 and white arrows for chromosome 7). On mitotic chromosomes, the telomere signals were detected on five pairs of chromosomes at both ends. In the case of dot chromosomes 5 and 7, the telomere signals were only detected at one end and another end was located by 25S rDNA (Fig. 6b, red and white arrows). In addition, interstitial telomere repeat sequences (ITRs) existed on mitotic chromosomes 1–4. In contrast, telomere repeats were only observed at the ends of all diakinesis chromosomes.

## Discussion

Various studies have shown that mulberry contains abundant polyploid levels and similar chromosome morphologies^4, 8^. These studies were focused on chromosome counting and chromosome behaviors during the meiotic phase. However, these works were carried out using traditional methods and most of the chromosomes were not traceable or distinguishable. In *M. notabilis*, which has 14 chromosomes of different lengths, two middle pairs of chromosomes and three pairs of dot chromosomes have still not been discriminated. Therefore, new techniques with higher resolution are needed for chromosome studies in these species. FISH has proved to be an important molecular cytogenetic technique and FISH-based chromosome identification can provide accurate karyotypes that can form the basis for phylogenetic analysis^24^.

The availability of the genome sequence of *M. notabilis* provided an opportunity to analyze the genome organization^13^. Single-copy sequences and repeat sequences can be identified and used as probes for FISH. Here, chromosome identification of *M. notabilis* was carried out by FISH using single-copy sequence probes and rDNA probes. All chromosomes were successfully identified. Prominent among these were three dot chromosomes easily ordered based on the signal patterns.

Meiotic karyotypes have been widely studied in plants^24^, especially pachytene karyotype. The pachytene chromosome contained four chromatids and was 10–40 fold longer than the metaphase chromosome^16^. The pachytene chromosome had a higher axial resolution and sensitivity in FISH and more morphology landmarks, which made it a better choice for cytogenetic studies. However, the well-spread and separated pachytene chromosome was difficult to prepare in most of the plants^25^, which we also found in *M. notabilis*.

Unlike the pachytene chromosome, the diakinesis chromosome contains homologous chromosomes that further condensed into bivalent or multivalent chromosome. The diakinesis chromosome has usually been used in polyploidy studies^23, 26^. Boldrini *et al*.^27^ used the diakinesis chromosome to estimate the basic chromosome number of *Brachiaria humidicola*. In this study, we obtained a systemic diakinesis karyotype of *M. notabilis* by analyzing chromosome morphology, relative chromosome length, and FISH signals (Figs 2 and 3). All the chromosomes were fully paired into bivalents with good sharps and more morphology landmarks, especially in diakinesis chromosomes 3 and 5. Chromosome 3 was clearly distinguished from chromosome 2 based on the FISH signal patterns and diakinesis chromosome morphology, which was impossible in mitotic chromosomes. Diakinesis chromosome 5 was easy to identify because it showed a unique morphology in which the long arm was thicker than the other arm (Fig. 2e).

According to the mitotic and diakinesis karyotype of *M. notabilis* described above, the 14 mitotic metaphase chromosomes showed seven distinct pairs and the diakinesis chromosomes showed six fully paired bivalents (discussed below). Recently, wild mulberry resources with chromosome numbers of 2n = 35 and 2n = 49 have been found by our team. These chromosome numbers are multiples of seven, not 14. Thus, the basic chromosome number of *Morus* is convincingly considered to be seven.

Polyploidy, dysploidy, and aneuploidy are thought to have played important roles in the karyotype evolution of eukaryotes^28, 29^. Dysploidy can cause increases or decreases in basic chromosome numbers through chromosome fission or fusion, respectively. Nested chromosome fusion that can cause basic chromosome number decrease have been reported recently^30^. However, all the plants used in these studies had already formed different basic chromosome numbers in their genus or species. Based on the synteny of chromosome segments, the pathway of karyotype evolution was reconstructed^31^. Here, the chromosome complement of *M. notabilis* showed only six chromosomes after the diakinesis stage, which was one less than the mitotic complement. To date, this has not been reported in any other plant species. In detail, chromosomes 5 and 7 maintained their separation from the leptotene stage to the zygotene stage (Fig. 4). These two chromosomes began to associate together from the diplotene stage to the pre-diakinesis stage, and two 25S rDNA signals existed for some time. During the diakinesis stage and the two meiosis stages, chromosomes 5 and 7 were fully associated, because only one 25S rDNA signal existed in the meiotic chromosome complement. In the somatic cells, some of the meiotic chromosome 5 s broke into mitotic chromosomes 5 and 7, and the mitotic chromosome number recovered to 2n = 14, although some of the somatic chromosome number 2n = 12 remained. Based on these signal patterns of the 25S rDNA probe, we proposed that mitotic chromosomes 5 and 7 fused or recombined into a slightly unstable diakinesis chromosome 5, and then the fused chromosome broke into chromosomes 5 and 7 in somatic cells. In short, this process suggested a chromosomal fusion–fission cycle in *M. notabilis* that might be mediated by rDNA.

The chromosomal fusion–fission cycle was first reported in field bean, *Vicia faba* (2n = 14), where one individual had one pair of metacentric chromosomes that broke into two pairs of telocentric chromosomes^32, 33^. This fusion–fission cycle was observed in only one seeding among 2096 progenies, where half the somatic cells had a chromosome number of 2n = 14, and the other half had a chromosome number of 13. Together with the species that contained a chromosome number of 2n = 12 and the morphology of the involved chromosomes, Schubert *et al*.^33^ proposed the fusion–fission cycle was reversible. However, in the present study, the fusion–fission cycle was observed between mitotic and meiotic chromosomes that existed in the same individual. This cycle is remarkably different from the cycle reported in *V. faba*. Therefore, the fusion–fission cycle is a novel one that has not been observed till now.

In this study, telomere signals were detected only on one end of the metaphase chromosomes 5 and 7 and no ITRs were observed on diakinesis chromosome 5, implying that the chromosomes 5 and 7 were fused at the ends without telomere signals. In combination with the signals of 25S rDNA on mitotic chromosomes 5, 7 and diakinesis chromosome 5, it seems reasonable to suggest that chromosome fusion occurred at the chromosome ends harbored 25S rDNA loci in *M. notabilis*.

In conclusion, we applied the molecular cytogenetic technique FISH in *M. notabilis*. The mitotic karyotype was constructed accurately and can act as the bases for future cytogenetic research in other mulberry species. The comprehensive FISH-based diakinesis karyotype was also built, and the chromosome information that was obtained will have a wide usage in other plants. The fusion–fission cycle of chromosomes 5 and 7 between the meiotic and mitotic phases in the same individual is reported here for the first time. This process suggests the natural basic chromosome number can be altered, and that the *M. notabilis* karyotype goes through this process.

## Materials and Methods

### Material and DNA preparation

The wild mulberry germplasm resource *M. notabilis* (2n = 14) used for all the FISH experiments was collected from a pristine forest in Ya’an, Sichuan Province, Southwest China (29°45.278′N, 102°53.878′E). The genomic DNA was extracted from *M. notabilis* leaves using the CTAB method^34^.

### Chromosome preparation

The mitotic chromosomes were prepared as described previously with minor modifications^13^. In brief, young leaves were pretreated with 2 mM 8-hydroxyquinoline at room temperature for 3 h, then fixed in 3:1 methanol/glacial acetic acid at 4 °C for 24 h. Fixed leaves were incubated with 0.067 M KCl solution for 1 h, then digested by 2.5% (W/V) cellulase Onozuka R-10 (Biosharp, Hefei, China) and 2.5% (W/V) pectolyase Y-23 (Biosharp, Hefei, China) at 30 °C for 3 h. Digested leaves were rinsed with ddH_2_O at room temperature for 1 h. For meiotic chromosome preparation, young inflorescences were fixed in 3:1 methanol/glacial acetic acid at room temperature for at least 24 h and stored at −20 °C before use. After removal of calyx, anthers from an inflorescence were incubated with 0.067 M KCl solution for 1 h, and then digested by 2.5% (W/V) cellulase Onozuka R-10 and 2.5% (W/V) pectolyase Y-23 at 30 °C for 7 h. Digested anthers were rinsed with ddH_2_O at room temperature for 1 h. The treated leaves and anthers were smashed into fine suspensions in 3:1 methanol/glacial acetic acid. Two drops of the cell suspensions were added onto a glass slide and dried by flame. The slides were stained with 4′,6-diamidino-2-phenylindole (DAPI) for 8 min. Then, the slides were screened under an Olympus IX73 microscope to select well-spread chromosome preparations. The remaining slides were dehydrated by baking at 37 °C for 30 min, then stored at −20 °C until use.

### Probe preparation

The *Morus* Genome Database (MorusDB) (http://morus.swu.edu.cn/morusdb) was used to screen the single-copy sequences^35^. Based on BLASTn searches against MorusDB, the repeat-free sequences of single-copy genes and the flanking regions >10,000 bp were selected. The repeat-free sequences (morus027496, morus027717, morus026579 and SSR2524) were amplified into lengths of 1,154~3,100 bp with primers designed using the Primer5 software^36^. The amplified sequences were cloned into a pMD19-T vector, and verified by both end sequencing. The purified PCR products of the clones were mixed equally, and then labeled with digoxigenin-11-dUTP using a DIG-Nick Translation Kit (Roche, Mannheim, Germany) according to the product manual. These probes were used directly for FISH. The primers for the 5S and 25S rDNAs were designed from the conserved sequences according to the multiple sequence alignment results obtained using the BioEdit software^37^. The primers for cloning Arabidopsis-like telomere were designed according to Ling *et al*.^38^. The 5S and 25S rDNA sequences were cloned into a pMD19-T vector, and then labelled with biotin-16-dUTP and digoxigenin-11-dUTP, respectively, using a PCR DIG Probe Synthesis Kit (Roche) with labeling conditions based on the product manual. The telomere sequences were cloned into pMD19-T vector and labelled with biotin-16-dUTP. The PCR cycles consisted of an initial denaturation of 95 °C for 5 min; followed by 32 cycles of 95 °C for 30 s, 58 °C for 30 s, 72 °C for 1 min; then 7 min for the final extension. The primers used in this study are listed in Supplemental Table S1.

### Mitotic chromosome FISH

Slides with well-spread chromosome preparations were baked at 37 °C for 2 h. To reduce the signal background, the slides were subsequently incubated in 100 µg/ml RNase at 37 °C for 1 h and in 1 µg/ml protease K at 37 °C for 15 min. The slides were washed in 2× standard sodium citrate (SSC), once at 37 °C for 5 min and twice at room temperature for 5 min. The chromosomes were denatured with 70% formamide in 2× SSC at 72 °C for 10 min, immediately dehydrated in 70%, 90%, and 100% ethanol for 5 min in each, and dried in air. The hybridization solution (80 µl of 50% formamide in 2× SSC, 10% dextran sulfate, 0.25% SDS, 125 ng/ml salmon sperm DNA, and 5 ng/µl probes) was denatured at 95 °C for 6 min, then chilled on ice for at least 10 min. Next, the chromosomes and hybridization solution were denatured together at 80 °C for 5 min. After overnight hybridization at 37 °C, the slides were washed with 10% formamide in 2× SSC at 37 °C for 10 s, twice in 2× SSC at 37 °C for 3 min, then in 0.2% Tween 20 in 4× SSC at room temperature for 3 min. The slides were blocked with 1× blocking solution (Roche) in 4× SSC at 37 °C for 20 min. The digoxigenin- and biotin-labelled probes were detected with 1ng/µl anti-digoxigenin-fluorescein, Fab fragments from sheep (Roche, Mannheim, Germany) and 1ng/µl streptavidin-Cy3 (Zymax Grade) (Invitrogen, CA, USA), respectively. After incubation at 37 °C for 1 h, the slides were washed with 0.2% Tween 20 in 4× SSC once at 37 °C and twice at room temperature for 3 min. Chromosomes were counterstained with 1 ng/µl of DAPI in darkness for 8 min, then washed with 2× SSC at room temperature for 3 min. Finally, the slides were covered with cover slips and sealed using nail polish.

### Meiosis chromosome FISH

The meiosis chromosome FISH was carried out using the mitotic chromosome FISH protocol described above with the following modifications. The digestion times of RNase and protease K were lower, namely 30 min and 5 min, respectively. To fix the chromosome, two steps were added before the chromosomes were denatured with 70% formamide; i.e., the chromosomes were fixed with 4% formaldehyde at room temperature for 10 min and then dehydrated in 70%, 90%, and 100% ethanol for 5 min in each.

### Image capture and analysis

All chromosome images were captured with an Olympus IX73 microscope using the cellSens Standard 1.13 software and a CCD camera DP80 in grayscale channels. The images were then pseudocoloured with the cellSens Standard 1.13 software. Finally, the images were adjusted using Adobe Photoshop CS6 and merged by ImageJ software^39^. To construct the karyotypes of *M. notabilis*, each chromosome pair was separated from the images with chromosomes identified. The relative lengths of the diakinesis chromosomes were measured using ImageJ software in 30 cells^39^.

## Electronic supplementary material

Supplementary Information
